# Mechanisms, Economy, and Performance of Advanced Footwear Technology in Endurance Running—A Review

**DOI:** 10.3390/muscles5010002

**Published:** 2025-12-24

**Authors:** Daido Dagne Bruvere, Edgars Bernans

**Affiliations:** 1 Latvian Academy of Sport Education, Riga Stradins Univeristy, LV-1007 Rīga, Latvia; daido.bruvere@rsu.edu.lv; 2 Sports Heathcare Research Center, Riga Stradins Univeristy, LV-1007 Rīga, Latvia

**Keywords:** running shoes, advanced footwear technology, carbon-plated shoes, running biomechanics, running economy, running performance

## Abstract

Since the introduction of advanced footwear technology (AFT) in 2017, numerous world records from 5 km to the marathon have been broken. Among these innovations, carbon-plated shoes have received particular attention. Previous research indicates improvements of 2–4% in running economy (RE), which translates into an approximate 1–2% improvement in running performance when running in these shoes. The rapid progression of performance has generated significant scientific interest; however, a clear understanding of the mechanisms driving the effectiveness of AFT remains limited. Despite widespread adoption and remarkable results, the mechanisms underlying the effectiveness of AFT are still not fully understood, which is why optimising its potential benefits continues to be an ongoing challenge. This review summarises current knowledge on AFT and critically evaluates the biomechanical and physiological mechanisms underlying their effects on RE and performance. It also highlights the interaction between shoe design features and individual biomechanics, supporting evidence-based approaches to footwear selection and training strategies tailored to athletes’ needs. A clearer understanding of these mechanisms may provide valuable insights for researchers, coaches, and athletes and help maximise the potential benefits of AFT.

## 1. Introduction

Running shoes can be categorised into several types based on their function and intended use, including minimalist, conventional, carbon-plated, motion control, support, and other types [1,2]. All of these can influence running economy (RE) and performance in long-distance runners [3,4,5] and may also alter running biomechanics [6,7,8,9].

In recent years, increasing attention has been paid to carbon-plated shoes—running shoes with an embedded carbon fibre plate in the midsole combined with multiple layers and specialised foam structures [10,11]. There remains some ambiguity regarding how to categorise these shoes. In the literature, terms such as “plated shoes”, “4% shoes”, “super shoes”, “super spikes”, “neoteric shoes”, “carbon shoes”, and “ergogenic shoes” are used [1]. In this review, we refer to this category collectively as advanced footwear technology (AFT), with carbon-plated models representing the most widely recognised type.

Introduced by Nike in 2016 [12] and commercially available since 2017 [13], AFT has gained significant scientific and public interest due to its potential to enhance RE [10,14,15] and endurance race performance [16,17,18]. The growth in performance, especially in female athletes [19,20,21,22,23], has led some to compare AFT to the barefoot and minimalist running era of the 2000s [1].

Although a growing number of studies have analysed different brands and models of AFT, there is still no clear consensus on the mechanisms that explain their effectiveness. Moreover, questions remain about how these shoes can be optimally applied to individual athletes, considering biomechanical and physiological variability [24,25]. A clear understanding of AFT mechanisms is crucial both to optimising athlete-specific performance outcomes and to informing regulations that ensure fairness as footwear technologies continue to advance. This raises broader questions: How far will technological advances go? How will they influence the evolution of performance outcomes and the nature of sport? Could they even be considered a form of “technological doping” [26]?

The purpose of this review is to summarise and critically evaluate the dominant factors determining the working mechanisms of AFT, highlighting their role in shaping RE and performance. It further identifies key considerations for future research design and interpretation and provides practical insights for coaches and athletes on effective AFT use tailored to individual needs. The ultimate goal is to support the development of evidence-based and practically applicable guidelines for the assessment and use of AFT in sport.

To ensure comprehensive coverage of the literature, we conducted a broad narrative search across multiple databases, including PubMed, Scopus, Web of Science, and ProQuest. The primary focus was on publications from the past five years to capture the most recent developments, while earlier studies were included to illustrate the underlying mechanisms of advanced footwear technology (AFT) and its historical evolution in running footwear. Searches were conducted using combinations of key terms such as running AND shoes, running shoes, advanced footwear technology AND running economy, carbon-plated shoes, advanced footwear technology AND running biomechanics, and advanced footwear technology AND running performance. Only articles published in English were considered. Studies were selected based on their relevance to running economy, biomechanics, and performance. The final synthesis integrates findings from experimental, observational, and review papers to provide a balanced overview of current knowledge and its practical implications.

## 2. The Evolution of Advanced Footwear Technology

Although AFT is commonly associated with recent decades, carbon fibre plates were already used in running shoe construction in the 1980s [27]. At that time, the concept was discussed under the term “energy return shoes” [28,29]. Early models such as Brooks’ Fusion and Fission and later Fila’s racing shoes embedded flat carbon plates in the midsole, but a more significant technological shift occurred when Adidas introduced a curved geometry-specific carbon fibre plate (ProPlate), which advanced the principle of bending–stiffness manipulation of the midsole. This design was associated with Haile Gebrselassie’s 2007 marathon record of 2:04:26 [27].

The modern era of carbon-plated footwear began in 2016 (commercially available from 2017) when Nike released the ZoomX Vaporfly 4% [12]. The name reflected the manufacturer’s claim of up to 4% improvement in RE, which was later supported by independent studies [10,14,15]. Vaporfly 4% incorporated a carbon fibre plate embedded in the midsole together with other design elements intended to increase bending stiffness [11] and reduce movement in the metatarsophalangeal joint (MTPJ), facilitating more efficient energy transfer [30] and contributing to forward propulsion, often described as a “springboard effect” [31].

Subsequent innovations, including Nike Air Zoom AlphaFly Next %, added features such as “Air pods,” intended to return up to 90% of stored energy according to the manufacturers—technological extensions rather than conceptual departures. Wearing this model, Eliud Kipchoge broke the 2-hour marathon barrier in 2019 during the INEOS 1:59 Challenge, although the result was unofficial under World Athletics regulations [32]. More recently, the evolution of these design principles continued with Nike Alphafly 3, used by Kelvin Kiptum Cheruiyot to set the 2023 world record of 2:00:35.

Significant improvements in performance and multiple record-breaking results have led other companies to adopt similar carbon-plate and high-energy-return midsole technologies. As a result, nearly all major brands, including Nike, Adidas, Asics, New Balance, Saucony, and Hoka One One, now incorporate these design principles into their racing footwear, and technological development continues to accelerate [33]. This historical progression of design principles directly informs ongoing scientific debates about the mechanisms underlying AFT effectiveness and their implications for performance and regulation.

## 3. Regulatory Responses to Advanced Footwear Technology in Elite Athletics

Opinions on the rapid development of AFT and its impact on performance are divided. On one hand, AFT can be viewed as a technological advancement that enables elite athletes, whose physiological limits are already near their peak, to further enhance performance. On the other, concerns have been raised about fairness and whether such external interventions align with the fundamental nature of sport, which traditionally centres on human physiological and technical abilities.

In the early stages of AFT development, not all athletes had equal opportunities to access these models. Initially, prototypes were provided only to select elite runners in high-profile competitions, creating potential inequalities. Even after their public release, some athletes continued to have access to the latest prototype models before they became commercially available, leading to situations where runners switched sponsors to obtain perceived performance advantages [31].

To address these concerns, World Athletics introduced regulations in 2020 governing the use and design of AFT in elite competitions [34]. According to the updated competition technical rules [35,36], the sole thickness of AFT shoes must not exceed 40 mm (Table 1). Previous studies have suggested that even relatively small changes in stack height within a 20 mm range can meaningfully affect performance [37]. In addition, the shoe must not contain more than one carbon fibre plate, or any other material with similar properties, regardless of whether it spans the full length of the sole or only part of it [36]. These restrictions do not apply, however, to the National Collegiate Athletic Association or high school athletics, where all types of running shoe construction remain permitted [38].

For athletics spikes, regulations allow an additional carbon plate only for attaching the spikes, and the sole thickness must not exceed 30 mm [36]. To uphold fairness, World Athletics also mandated that all innovative shoes released after 30 April 2020 must be commercially available to all athletes for at least four months before a relevant competition. Shoes not meeting this requirement are considered prototypes and are prohibited in competition [36].

These regulations were designed to safeguard equality and fair play while establishing reasonable boundaries for technological innovation in sport-related performance enhancement. They also clearly reflect ethical considerations of technological fairness, ensuring that access to innovation does not compromise the integrity of competition.

## 4. Design Elements of AFT

**Figure 1:**
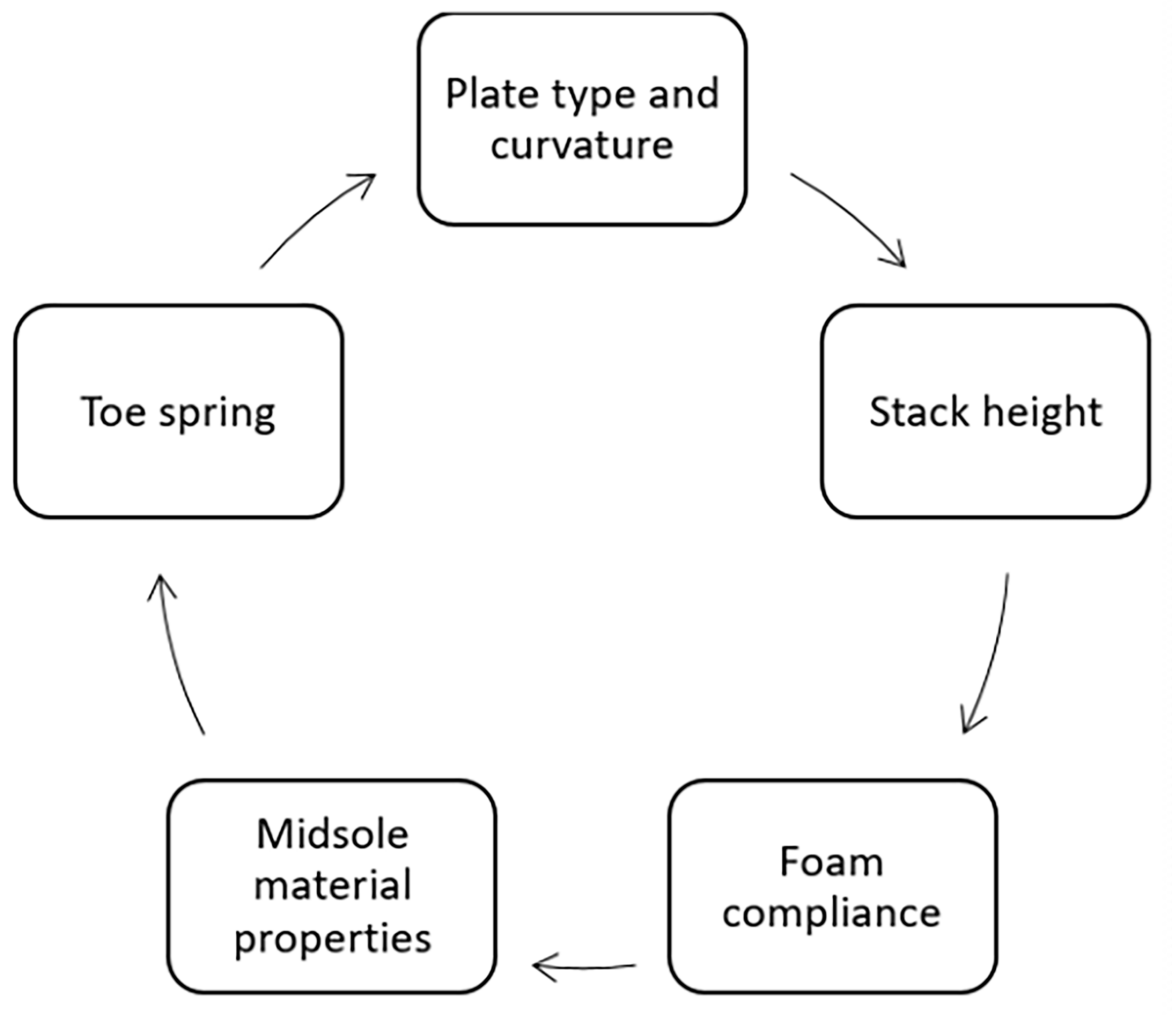
Conceptual schematic illustration of the key design elements of AFT and their interrelations.

The construction of carbon-plated shoes can be conceptualised as a layered structure, often described as a “sandwich”, consisting of multiple layers of varying types and thick- nesses compressed together [17]. Each of the elements—plate geometry, foam properties, longitudinal bending stiffness, toe spring, and stack height—has its role and should be evaluated in terms of their multi-factorial interactions (Figure 1). In the following section, the main design components of AFT and their interdependence will be discussed.

### 4.1. Plate Geometry and Midsole Material Properties

Usually defined as a “key structural element” of AFT, the carbon fibre plate [1,17] is either embedded within the shoe midsole or inserted directly into the shoe [39]. These two placements differ significantly. Insertable carbon soles, not surrounded by cushioning foam, are stiffer and may alter both the running feel and perceived comfort [40,41]. However, they tend to last longer and may be a more economical option than purchasing a new pair of carbon-plated running shoes [42].

Curved carbon plates are generally associated with greater improvements in RE and performance compared with flat plates [43,44,45,46,47,48,49].

A distinctive feature of many AFT models is rocker geometry, characterised by a slight forefoot elevation (“toe spring”) that creates an S-shaped sole design, in which the heel sits slightly higher than the forefoot. In interaction with increased midsole longitudinal bending stiffness (LBS), this configuration has been consistently associated with improvements in RE [9,40,42,43,50,51]. However, recent research indicates that the benefits may be runner-specific [52,53], and an “optimal shoe bending stiffness” may exist for different individuals [54]. In addition, some evidence suggests potential injury-prevention benefits due to increased stability and support for the foot and ankle joints [47].

The specific feature “toe spring” facilitates a faster and more efficient heel-to-toe transition, often described as a “rollover” or “spring-like” sensation, which helps to maintain a straighter alignment of the hallux, reduces energy expenditure during push-off, and shortens ground contact time [9,14,44,47,55]. In contrast, flatter shoe soles require greater muscular force and energy to complete this transition, leading to less efficient rollover mechanics [56].

Furthermore, it may help reduce overuse injury risk by decreasing peak pressure on the forefoot without increasing loads in the metatarsal region and by lowering demands on the ankle plantar flexors compared with flat-plate or non-plated shoes [45,47,48]. Mechanically, this effect is explained by the "teeter–totter" mechanism, in which the stiff carbon plate and curved sole act as a lever to reduce muscular effort during ankle dorsiflexion and push-off [24,37,56].

However, the design must be optimised to ensure that the teeter-totter effect occurs at the correct location (heel of the foot), time (push-off), and frequency (determined by running velocity and ground contact time) [56,57]. According to Nigg, Cigoja, and Nigg [56], three conditions must be met to achieve the effect:

1. Sufficient sole stiffness to shift the ground reaction force forward during stance.
2. Proper pivot point placement, ensuring that it is not positioned too far forward, so that the heel can act as a support point.
3. Appropriate forefoot curvature, enabling effective lever action and smooth rollover mechanics.

As noted by Willwacher et al. [58], translating these theoretical mechanisms into real-world running conditions is complex. Nonetheless, they provide valuable insights into critical design features that influence the effectiveness of AFT.

Plate location is also an important factor, particularly in models with increased stiffness [41]. The design of the forefoot plate—whether full-length or segmented—can substantially alter running biomechanics and, consequently, performance outcomes [59]. In contrast, inappropriate plate curvature or stiffness may increase the risk of foot injuries [41,47,51].

### 4.2. Foam Construction

Foam construction has been shown to play a particularly important role when interacting with carbon fibre plates [33,60,61] and may also influence shoe durability depending on the foam’s microstructure [62].

Aimar et al. [62] compared five commercial midsole foams derived from three of the most commonly used polymers in carbon-plated shoes—ethylene-vinyl acetate (EVA), polyether block amide (PEBA), and thermoplastic polyurethane (TPU) [60]—plus one modified sample obtained from an additional insert of the same midsole to capture structural variability. Under mechanical fatigue testing, EVA foams reinforced with microfillers demonstrated improved mechanical strength but reduced rebound properties and accelerated wear, likely due to weak cohesion between fillers and the polymer matrix. In contrast, denser foams with hierarchical microstructures exhibited slower damage progression and enhanced durability; however, they were associated with poorer initial mechanical properties.

Lloria-Varella et al. [63] examined whether shoe wear and degradation after a fatiguing trail run influenced biomechanics and whether switching to a fresh pair of shoes could restore mechanics altered by worn footwear. After the race, the participants’ own shoes showed reduced midsole thickness and increased stiffness, confirming degradation. Running mechanics also changed: contact time and step frequency increased, while flight time and tibial peak-to-peak acceleration amplitude decreased. Contrary to their hypothesis, replacing the worn shoes with new ones did not significantly alter the main biomechanical variables, although it did affect shoe × time interaction.

### 4.3. Stack Height

Foam construction in modern AFT serves not only to provide additional cushioning but also to increase the stack height of the shoe [60,61]. Stack height is a critical design parameter, as it determines the thickness of the midsole and, consequently, the shoe’s capacity to store and return mechanical energy. Functionally, stack height acts like a compliant spring: a thicker midsole allows for greater elastic deformation during foot strike (via sole compression) and subsequent energy release during push-off, assisting propulsion [14]. This mechanism not only influences RE and biomechanics but also affects comfort and impact attenuation [37,61,64,65,66]. It may also contribute to fatigue resistance and reduced muscle damage or soreness over longer distances [16,67].

When comparing the Nike Zoom Vaporfly prototype (heel height: 31 mm; forefoot height: 21 mm) with two similar-technology shoes—the Adidas Adios Boost and the Nike Zoom Streak 6—Hoogkamer et al. [14] reported that the prototype deformed nearly twice as much (11.9 mm; energy return: 87%) compared with the Adios Boost, with 6.1 mm (75.9%), and the Zoom Streak 6, with 5.9 mm (65.5%).

A recent study by Baumann et al. [61] confirmed that adding an additional 10 mm of stack height to the current 40 mm limitation improved running economy (RE) by 0.6% during treadmill running and 0.7% during overground running. Interestingly, this modification did not significantly affect perceived exertion ratings or running kinematic variables (step frequency, flight time, ground contact time, duty factor, etc.). Moreover, the shoes with the highest stack height (50 mm) were rated the lowest in terms of subjective comfort. One possible explanation for this could be the reduced running stability, primarily resulting from lateral instability associated with greater ankle eversion—a factor previously linked to an increased risk of injury [37,65,66,68,69], particularly among runners with pre-existing foot conditions or excessive pronation [65]. However, evidence supporting this remains limited, and further research is needed.

## 5. Working Mechanisms of AFT

Understanding the underlying working mechanisms of AFT is essential to explaining the improvements in RE, biomechanics, and performance observed in recent years. The interaction of shoe geometry, material properties, and runner-specific biomechanics determines how effectively mechanical energy is stored, transferred, and returned during the gait cycle. Collectively, it illustrates that the effectiveness of AFT arises from various multiple-element interactions. A deeper understanding of it may contribute to more effective performance outcomes.

### 5.1. Energy Return Mechanisms of Running Footwear: Implications for AFT Performance

The storage and release of elastic energy is recognised as one of the main factors contributing to RE in both humans and animals. When analysing the role of mechanical energy in performance, three strategies are commonly identified [43,58]:

1. Optimisation of musculoskeletal function.
2. Enhancement in energy return.
3. Reduction in energy expenditure.

Although the first factor has received less attention, the latter two strategies are highly relevant to AFT development, where material composition and mechanical properties are manipulated to maximise performance benefits [58,70]. The primary function of the energy return mechanism in footwear is to recover energy stored in the midsole during stance and return it to assist subsequent movement, such as the swing phase. This process is associated with reduced oxygen consumption and improved RE [71,72].

#### The Role of the Metatarsophalangeal Joint (MTPJ) in Energy Return Mechanisms

The metatarsophalangeal joint (MTPJ) plays a central role in running efficiency, as its motion strongly influences mechanical energy transfer and potential energy loss during the push-off phase. Excessive bending motion at the forefoot or MTPJ can increase mechanical energy loss during running, sprinting, and jumping [57,73]. Carbon fibre plates are designed to limit excessive MTPJ dorsiflexion, thereby reducing energy dissipation and enhancing effective energy transfer [30], which is generally associated with improved RE [9,40,42,43,50,58]. Joint mechanics can also be modulated by changes in stiffness and elasticity of surrounding structures, further highlighting the importance of the MTPJ in footwear design [57].

In a study of shoes with varying sole stiffness, Willwacher et al. [58] observed that in the stiffest models, the centre of pressure shifted significantly toward the forefoot during the final 40% of stance. Furthermore, the authors proposed four strategies to optimise the runner–shoe interaction at the MTPJ:

1. Prolong the propulsion phase to increase plantar flexion work, as positive work is mainly generated at the end of propulsion.
2. Increase longitudinal bending stiffness so dorsiflexion occurs earlier, allowing more time for plantar flexion and greater positive work.
3. Modify shoe construction (e.g., toe spring) to initiate dorsiflexion earlier; reducing excessive forefoot curvature may improve rollover mechanics and efficiency.
4. Optimise extrinsic muscle conditions by improving force–velocity characteristics.

Beyond these strategies, effective energy return depends on precise timing and location of sole flexion, ideally aligning plantar flexion with toe-off. The optimal scenario occurs when both midsole and toes perform plantar flexion at the end of propulsion, transmitting energy at the right time and place. Maximum benefit would occur if the flexion–extension cycle is fully realised within ground contact [57,58]. However, achieving such ideal conditions is unlikely. Footwear stores and returns far less elastic energy than tendons, with up to 30% being lost in the process [57,58]. Thus, the primary advantage of AFT lies in reducing energy dissipation rather than truly increasing energy return [43]. Accordingly, footwear development should prioritise strategies to minimise energy loss rather than attempting to maximise energy return.

### 5.2. Materials and Mechanical Properties of Carbon-Plated Shoes

Recent studies indicate that the type of sole material and its mechanical properties may have a greater impact on RE, efficiency, and fatigue resistance than longitudinal bending stiffness (LBS) [15,16,50,62,74].

Resilience (the ability to return part of the stored mechanical energy) and compliance (the deformation that occurs under a given force during compression) are identified as key properties in the design of AFT [14,72,75,76,77]. Running shoes with more resilient and compliant midsoles can reduce adenosine triphosphate (ATP) consumption during muscle contraction, producing the same force with lower oxygen cost at any running intensity [15]. The most common midsole materials used in AFT are ethylene-vinyl acetate (EVA), thermoplastic polyurethane (TPU), and polyether block amide (PEBA) [60]. Among them, PEBA is considered one of the most efficient materials for energy return [14,15]. Its low density allows for greater cushioning volume without significantly increasing shoe mass [14,60]. Despite these favourable mechanical properties, PEBA’s low density results in faster wear compared with conventional materials. This compromises both durability and long- term impact on RE. Rodrigo-Carranza et al. [60] found that while new PEBA shoes improved RE more than EVA shoes, after 450 km of use, the two materials had similar effects. Moreover, PEBA showed a larger increase in energy consumption (0.32 ± 0.38 W/kg) than EVA (0.06 ± 0.58 W/kg) when comparing new and worn shoes. These findings suggest that although highly effective in active use, PEBA midsoles have low durability, leading many runners to reserve carbon-plated shoes for races and high-velocity training sessions.

In summary, an optimal carbon-plated shoe design should combine lightweight construction, effective traction, and compliant, resilient foams [14,62,78].

## 6. Athletic Spikes and “Super Spikes”

The development of athletic spikes has mirrored that of AFT, with the term “super spikes” now commonly used alongside “super shoes” as a product of AFT innovation.

AFT-based athletic spikes first appeared in competition in 2019, with Nike introducing the first widely recognised model, the Nike ZoomX Dragonfly [38,74,79]. Today, super spikes are used by the majority of elite athletes in major competitions [17,38]. The Nike ZoomX Dragonfly and Nike Air Zoom Victory remain among the most prominent models, though manufacturers such as Puma are rapidly advancing their own designs [80].

In terms of construction, super spikes share many features with super shoes, including stiff curved carbon plates and lightweight, resilient foams with high energy return. Some models also incorporate additional technologies such as Air Units [17,74]. However, performance improvements reported for super spikes (typically 1–2%) remain slightly lower than those observed for carbon-plated road shoes, despite the spikes’ lower mass [2,15,17,64,79,80]. One possible explanation is the reduced midsole thickness of super spikes (20–25 mm compared with up to 40 mm in AFT road shoes), which may limit energy storage and return [14,33]. However, as stack height alone may not determine performance outcomes, further research is needed.

Testing spikes under controlled laboratory conditions is also difficult. In treadmill experiments, spikes are often removed, altering shoe mass and mechanical function, which can influence measured effectiveness [38,81]. Another problem is that the energy demands of middle-distance track events (e.g., 1500 m), which rely mainly on the anaerobic energy system, cannot be directly transferred to the half-marathon or marathon, which makes it difficult to quantify the exact benefits from super spikes [82]. To address these challenges, Bertschy et al. [80] compared three middle-distance super spike models with traditional spikes in 12 competitive middle-distance runners, at their 800 m or 1500 m race paces. They also included a Puma prototype with 19 mm Nitro foam and a carbon sole. Running with super spikes increased velocity by 1.6–2.1% compared with traditional spikes, with the Puma prototype showing the greatest improvement (up to 3.1%), outperforming the Nike Dragonfly even under varied conditions. These improvements were attributed primarily to biomechanical changes, particularly ~2% longer stride length in super spikes versus traditional spikes.

Collectively, these findings suggest that super spikes are more economical than traditional athletic spikes, although the mechanisms remain unclear. Despite their lighter mass, super spikes still fall 1–2% short of the performance improvements observed in AFT road shoes. This is primarily due to reduced cushioning, which highlights the trade-off between mass reduction and energy dissipation. Future research should focus on identifying design modifications that could narrow this gap while preserving the mass advantage, paving the way for the development of next-generation spike technology.

## 7. Effects of AFT on Running Biomechanics

Evidence suggests that AFT primarily influences running kinematics rather than physiology [11,12,42,65,83]. These effects are reflected in changes in spatio-temporal gait parameters, including step length, step frequency, contact time, and flight time. Runners adapt their movement patterns depending on footwear [12,84,85] and running surface [75,86,87,88,89]. Such adaptations can substantially affect metabolic measurements [90], given the close relationship between running biomechanics and RE. Importantly, changes in kinematics may improve, impair, or have no significant effect on RE [91].

### 7.1. Working Mechanisms of Carbon-Plated Shoes

Carbon-plated shoes influence running biomechanics by modifying force transmission through the foot–shoe–ground interface. The embedded carbon fibre plate acts as a stiff lever, resisting midsole bending and altering ankle and metatarsophalangeal (MTP) joint mechanics [92,93,94]. By limiting excessive dorsiflexion at the MTP joint during toe-off, the plate reduces energy loss in this region [30,70] and promotes a more efficient forward roll of the foot—often referred to as the “teeter–totter effect” [24,56].

This stiffening effect shifts the point of force application anteriorly, increasing the lever arm of the ground reaction force and thereby altering joint moments at the ankle and knee. In combination with compliant, resilient foams, the carbon plate enables elastic energy storage during loading and contributes to energy return during push-off [14,17,38,58,65,95]. Ideally, these mechanisms promote a more even distribution of impact forces across the foot, reducing excessive load on the MTP region and lowering the energetic cost of running [44,47,96].

Recent studies indicate that these effects are further optimised with a curved carbon plate, which smooths transition through stance and reduces localised forefoot stress compared with a flat plate [44,45,47,48]. This design may be particularly important in long-distance running, where sustained loading increases injury risk. By reducing forefoot stress and supporting smoother roll mechanics, curved carbon plates may contribute to both injury prevention and fatigue resistance [16,47,67].

### 7.2. AFT and Running Injuries: Altered Biomechanics Theory and Practical Examples

Changes in running biomechanics caused by AFT can sometimes produce inadequate sensations for athletes. Increased midsole cushioning, common to nearly all AFT models, allows for reduced knee flexion [86]. This encourages more rearfoot striking without the runner perceiving the same impact as in conventional shoes or, even more so, barefoot running [78,97].

Theoretically, this should enable runners to sustain higher speeds and longer distances with less fatigue, a notion supported by studies reporting reduced fatigue, muscle damage, and pain when using carbon-plated shoes during both interval training (5 × 1000 m with 90 s recovery) [16] and marathon running [67]. However, this explanation is overly simplistic. The absence of immediate discomfort does not mean that bones, joints, and ligaments remain unaffected. A recent study by Baumann et al. [61] indicated a potential increase in lower-limb loading when running in AFT. Over time, the passive musculoskeletal system may struggle to tolerate and recover from altered loading, increasing the risk of overuse injuries [25,61]. Increased heel striking could also elevate the risk of rearfoot injuries, particularly when running on stiff surfaces [78,90,97].

Although direct evidence is limited, case reports describe stress reactions and stress fractures of the midfoot (navicular bone region) in athletes using carbon-plated shoes at the time of injury [51]. These cases involved individuals of varying age, sex, and sport, including triathlon. Some had a prior history of stress-related bone injury in the same region. Incomplete data on biomechanics during injury limit causal inference, but the observations suggest a potential risk associated with AFT use. Diagnosis of stress fractures typically requires weeks or months, complicating the interpretation of injuries appearing soon after AFT use [94].

Overall, while carbon-plated shoes clearly influence running biomechanics, the precise mechanisms and shoe features responsible for both performance gains and potential injury risks remain unclear—an issue highlighted in recent AFT research [24]. Individual factors such as training background, injury history, and biomechanical characteristics may determine whether a shoe model is beneficial or harmful [25,98].

Gradual integration of AFT into training is required. This approach allows runners to adapt their musculoskeletal systems and gait patterns, while reducing potential overuse injuries and optimising the advantages of advanced footwear technology [93,98]. Nevertheless, despite the benefits provided, the long-term effects of AFT should be carefully considered, as it is unclear what impact these shoes have on the musculoskeletal system, natural gait patterns, and running stability indicators.

## 8. Effects of AFT on Running Economy and Performance

Running economy (RE) reflects the oxygen cost at a given submaximal velocity and is widely regarded as one of the most important performance determinants in long-distance running [11,33,99]. Despite extensive study, findings on AFT remain inconsistent. Reported outcomes range from significant improvements to no measurable effect [8] or even negative impacts on RE and performance [51]. Placebo effects have also been proposed [100] and were recently demonstrated in recreational runners [101].

Many studies have reported ~4% improvements in RE with AFT [9,10,14,15,17], consistent with the performance claims of Nike’s first carbon-plated model—the ZoomX Vaporfly 4% [12,22]. Translating these gains into race performance suggests improvements of ~1–2% [19], equivalent to ~79 s in a world-class men’s marathon, based on the estimate that each 1% increase in velocity shortens finishing time by ~79 s [102].

Several studies have highlighted the positive impact of carbon fibre soles on RE [29,32,33,103]. Investigations of the Vaporfly and Alphafly have demonstrated faster times in controlled 3 km and 5 km trials [50]. Hoogkamer et al. [14] reported an average 4% improvement in RE (reduced oxygen consumption) at 14–18 km/h when running with a Vaporfly prototype compared with Nike Zoom Streak 6 flats and Adidas Adios BOOST 2 shoes, despite equalised shoe mass (+51 g). This was widely linked to the sub-2-hour marathon barrier, which Kipchoge later broke while wearing the Vaporfly Next% [31,32]. Barnes and Kilding [15] confirmed similar benefits at matched speeds. In contrast, Healey and Hoogkamer [8] cut the Vaporfly’s carbon plate and found no significant change in RE (−0.55 ± 1.77%), suggesting that the plate alone does not explain performance benefits.

AFT outcomes appear to depend on multiple interacting factors. Internal influences include individual responses to specific shoe models [25,52], training level and genetics [5], and biomechanical, physiological, and anthropometric characteristics [24,43,98,104]. External factors include surface stiffness [90,105], running velocity [9,14,15,16,18,40,79], race distance [11,42,106], and shoe mass [78,107,108]. Design features such as midsole bending stiffness [30,54,109], foam compliance and resilience [60,62,65], plate type and curvature [43,44,47,56,110], and stack height [37,61,64,66,69] could further modulate outcomes [2,18,38].

To reliably capture these effects, methodology must account for device variability. Measuring RE requires at least two same-day trials, as day-to-day variability in metabolic systems can reach 14%, producing coefficients of variation in RE from 0.3–8.5% [111,112]. Averaging same-day measurements, therefore, provides more stable values, at least in moderately trained male runners.

Collectively, these findings indicate that AFT-related improvements in RE and performance arise from multiple interacting factors rather than any single feature such as the carbon plate [8,104].

## 9. The Main External and Internal Factors Influencing the Working Mechanisms of Advanced Footwear Technology

**Figure 2:**
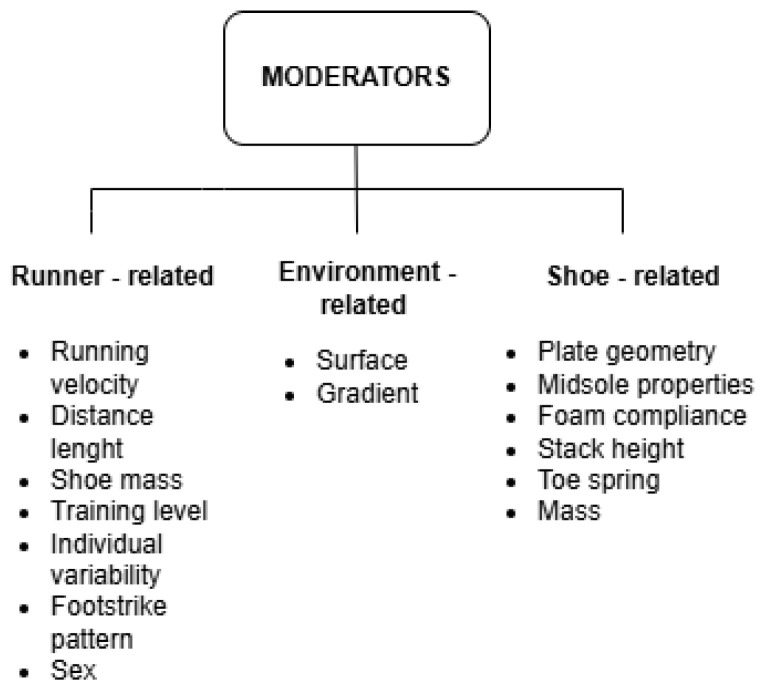
A map of the moderators of the key internal and external factors influencing the working mechanisms of Advanced Footwear Technology (AFT), divided into categories.

Understanding AFT requires examining not only its design, but also the internal and external factors determining its effectiveness. Careful analysis of these variables is equally important for coaches and athletes choosing footwear and for researchers refining methodologies to produce more objective and applicable evidence. The present section summarises and deeply analyses the main internal and external factors (Figure 2) influencing the working mechanisms of AFT, tailored to optimise the performance-maximisation strategies according to individual needs and characteristics.

### 9.1. Running Velocity

Running velocity strongly influences the effective realisation of AFT benefits. Nearly all previous studies have observed differences in RE and performance across velocities [14,15,40,113].

The observed differences are largely explained by velocity-dependent changes in running kinematics (stride length, frequency, vertical oscillation, etc.), which are known to vary substantially between lower and higher running speeds [114]. At lower velocities, the biomechanical conditions necessary to fully exploit AFT benefits may not be achieved, limiting the “shoe potential,” since AFT is primarily designed for racing applications.

Day and Hahn [40] confirmed this by comparing conventional shoes with models of increased and very high stiffness at velocities of 14, 17, and 20 km/h. RE improved significantly at 17 km/h in the stiffest shoes, despite being 50 g heavier than the control model—a weight difference too small to meaningfully affect RE [14,103]. Participants also reported greater comfort at higher velocities in stiffer shoes, supporting the hypothesis that velocity-dependent biomechanical changes, particularly at the ankle and MTPJ, influence AFT function [30,92,93].

The greatest RE improvements (~4%) have been reported at submaximal intensities of 14–18 km/h, corresponding to typical race-pace velocities in well-trained endurance runners [14,15,33]. Barnes and Kilding [15] found that Nike Vaporfly shoes improved RE by 4.2 ± 1.2% and 2.6 ± 1.3% compared with Adidas Adios Boost 3 and Nike Matumbo 3 spikes at these velocities. Hoogkamer et al. [14] reported nearly identical improvements when comparing Vaporfly to Adidas Adios Boost and Nike Zoom Streak 6, defining RE as energy consumption (W/kg). In this case, percentage improvements were consistent across all velocities tested, with the largest benefits at 18 km/h. It is important to note, however, that even 18 km/h is ~13% slower than the average marathon world record pace (21 km/h), limiting direct extrapolation of these findings to world-class performance. Future research should focus on elite marathoners, who already operate at exceptionally high RE.

### 9.2. Lower Intensities

Runner training level and baseline (“natural”) RE, which differ between recreational and elite athletes, can strongly influence outcomes [5]. Nonetheless, improvements are also possible at lower intensities [38,95,113,115,116].

Joubert, Dominy, and Burns [95] tested runners at 10 and 12 km/h in conventional and carbon-plated shoes, observing RE improvements of 0.9% and 1.4%, respectively, compared with conventional models. Similarly, Bolliger, Spengler, and Beltrami [116] reported significant reductions in oxygen consumption and heart rate (*p* < 0.001) in recreational runners wearing Cloudboom Echo 3 AFT shoes versus conventional and prototype models of the same brand, with no clear velocity-dependent effect.

Other studies have not found significant biomechanical changes at lower intensities. For example, Bolliger et al. [116] observed no significant differences in step frequency, flight ratio, or leg stiffness (cf. [11,14,117]). Interestingly, the most comfortable shoes were associated with higher oxygen cost, challenging the assumption that comfort directly improves efficiency [118,119]. Shoe material properties [60], mechanical behaviour [17,50], and design features [62] may also be important determinants.

Hébert-Losier et al. [113] compared habitual (OWN), minimal (FLAT), and Nike Vaporfly 4% (VP4) shoes in 18 male recreational runners. Each runner completed three 1.5 km trials (1.1 km at a self-selected comfortable pace and 400 m at perceived 5 km race pace). Minimal biomechanical differences were found among the shoes, although FLAT produced higher step frequency and stiffness at slower velocities. VP4 reduced propulsion time and was perceived as more comfortable than FLAT, while OWN was rated the most comfortable overall and the least likely to cause injury. Comfort ratings appeared to depend more on individual perception than biomechanics, with running velocity strongly influencing perceptions.

Isherwood et al. [115] investigated sex differences across three AFT models. Both innovation and commercial models lowered oxygen consumption and improved subjective perception compared with another commercial model. Although female runners exhibited higher vertical loading rates and reduced joint motion, these differences did not affect subjective ratings. Both sexes benefited from AFT at moderate velocities, supporting its relevance for recreational populations.

Although the magnitude of improvement at lower velocities is smaller than that observed at higher intensities, these findings suggest that AFT benefits extend beyond professionals to amateur and recreational runners. However, a “critical velocity” may be required to fully activate AFT mechanisms. Runners unable to reach this threshold may experience limited benefits and potentially increased injury risk due to altered biomechanics.

### 9.3. Distance Length

Physiological and biomechanical differences across running distances are well established [11,39,114]. Although carbon-plated shoes are primarily designed for long-distance events (5 km to the marathon), relatively few studies have examined their impact in runs longer than 10 min [11,42,94,106]. This is noteworthy, as evaluating effects over extended durations would more closely reflect the physiological, biomechanical, and perceptual conditions of actual race distances.

Kiesewetter et al. [11] analysed biomechanical and physiological responses during a 10 km run in three carbon-plated shoes (Puma Fast–FWD, Puma Fast-R, and Nike Vaporfly Next %), which differed in plate configuration and midsole properties but not mass. Significant biomechanical adaptations were observed, particularly in foot strike pattern and joint kinematics, suggesting style adjustments to reduce lower-limb loading. Despite these biomechanical changes (e.g., maximum angular velocity, eversion velocity, and heel strike angle), physiological measures such as heart rate and VO_2_ did not differ significantly. A likely limitation was the relatively low exercise intensity (70% VO_2_max), which may not have fully activated AFT benefits. In addition, runs were performed on separate visits, introducing day-to-day variability that may have reduced measurement reliability [42,120].

Milner et al. [94] examined the effects of carbon-plated shoes (Nike Vaporfly 2) on metatarsal loading during two 45-minute runs at 65% of heart-rate reserve (HRR). Running in AFT significantly lowered peak MTP joint flexion angle and peak axial force (both p<0.0001), while substantially increasing bending moments (p<0.001), with an average rise of approximately 59.9% compared with the control shoe (Nike React Infinity Run 3). Notably, time did not exert a statistically significant effect on changes in metatarsal loading across the prolonged runs. The study was limited by a small sample size (n=10 females), and the authors emphasised the need for further research to confirm these findings and to explore inter-individual variability.

Hoeft [106] conducted a similar study in which eight runners completed two 30 min treadmill runs in carbon-plated versus non-plated racing flats. Participants were blinded to running velocity, and performance was expressed as changes in pace and speed. Carbon-plated shoes increased velocity by 0.237 km/h, equivalent to ~3.5 s/km (≈1.5% performance gain). However, physiological parameters (e.g., heart rate, RER, RE, and RPE) showed no significant differences, although a non-significant 3.2% improvement in RE (*p* = 0.184) was observed during a submaximal warm-up.

Perrin et al. [42] tested 13 well-trained male runners in a half-marathon treadmill trial at 95% of the second ventilatory threshold, comparing high-longitudinal-bending-stiffness (HLBS; carbon-plated) versus standard-stiffness (SLBS; conventional) shoes. Six-minute constant-velocity runs (12 km/h) were performed before and after the half-marathon to assess energy cost and ankle plantar flexor muscle force. No difference in energy cost was observed during the half-marathon itself, though the HLBS shoes were marginally more economical (~1%) in the shorter pre- and post-trials. The HLBS shoes also induced a greater reduction in plantar flexor force (−20.0 ± 9.8% vs. −13.3 ± 11.0%, *p* = 0.048) and were rated less comfortable (−1.2 ± 1.5 Borg points during the run and −0.8 ± 1.3 after). Biomechanically, the HLBS shoes increased contact time and push-off duration while reducing step frequency, leg stiffness, and vertical stiffness.

These findings contrast with earlier reports suggesting that carbon-plated shoes reduce energy cost and mitigate fatigue [14,15,16,17,67]. A potential limitation was the specific shoe model tested (Kiprun KS 900), in which the carbon plate was inserted as an insole rather than embedded in the midsole, differing from current AFT designs. Furthermore, trials were conducted over two sessions separated by 2–4 weeks, introducing individual variability and metabolic measurement instability that can affect outcomes [111,112].

Recent findings by Madsen et al. [121] indicated that carbon-plated shoes consistently demonstrated improved RE and lower heart rate, blood lactate, and oxygen consumption values over an 80 min running session at 95% of the lactate threshold, compared with non-plated shoe models. However, the progression of these physiological markers over time remained similar between shoe types. These results suggest that AFT improves RE without altering fatigue-related physiological responses and that individual variability and spatio-temporal variables, such as contact and flight time, may play a key factor in performance outcomes.

Overall, these findings suggest that while AFT can alter running style, this does not always translate into improved RE. The duration of the distance is, therefore, an important factor, and further studies are needed to clarify the effects of AFT during prolonged running, particularly under fatigue conditions.

### 9.4. Running Surface

Different running surfaces exhibit varying stiffness levels, which influence shock absorption and thereby alter biomechanical patterns and potentially affect RE [24,75,86,87,88,89,90,105].

When conducting laboratory research, the mechanical properties of treadmill surfaces require careful consideration, as they can significantly influence metabolic and performance outcomes [89]. Motorised treadmill surfaces can increase, decrease, or have no effect on energy consumption, depending largely on their stiffness [86]. Some treadmill surfaces equipped with additional shock absorbers can store and return up to 12% of mechanical energy [75].

Smith, McKerrow, and Kohn [90] compared running on two treadmills (HP Cosmos vs. Quinton), which differed in surface stiffness by a factor of 4.5. The stiffer treadmill was associated with higher oxygen consumption, carbohydrate oxidation, heart rate, and perceived exertion, alongside lower fat oxidation. Similarly, Kerdok et al. [75] demonstrated that reducing surface stiffness by 12.5 times lowered the metabolic rate by 12% and increased leg stiffness by 29%, without notable changes in support mechanics (e.g., ground reaction force, contact time, stride frequency, stride length, and vertical displacement of the centre of mass).

These results suggest that surface stiffness affects RE primarily through adjustments in leg stiffness, which help maintain centre-of-mass dynamics. Softer surfaces promote straighter leg mechanics, reducing muscle activity and energy demand [75,122], likely due to enhanced shock absorption [14]. A similar principle applies to track surfaces with added amortisation (“fast tracks”), where athletes often show notable performance improvements [123].

#### 9.4.1. Treadmill and Overground Running in the Analysis of AFT: Comparable or Not?

Despite these surface-related effects, most AFT studies are conducted indoors on motorised treadmills, raising questions about external validity. Van Hooren et al. [88] compared treadmill and overground running, concluding that the two are broadly comparable but biomechanical differences—especially sagittal-plane kinematics at foot strike—must be considered. Benson et al. [89] further noted that while some metrics, such as running power [124], may be similar across conditions, treadmill data cannot fully capture outdoor running dynamics.

Such differences complicate comparisons between treadmill-based RE studies and real-world overground performance [24,89]. Future research should, therefore, prioritise overground testing using wearable sensors (e.g., IMUs), which have been validated for accuracy under field conditions [80,125].

#### 9.4.2. Uphill, Downhill, and Level Running Conditions

While most AFT research focuses on flat-surface running, fewer studies have addressed incline and decline conditions.

Whiting, Hoogkamer, and Kram [126] tested Vaporfly shoes at treadmill inclines of +3° and −3° (~5%) at 13 km/h (3.61 m/s), reflecting gradients typical of the Boston Marathon. Vaporfly reduced energy consumption across all conditions compared with controls, though savings were ~1% smaller uphill (2.82%) and downhill (2.70%) than on flat terrain. Foot strike patterns shifted to the forefoot uphill and to the rearfoot downhill, suggesting that shoe design (e.g., stack height ratios and midsole properties) may require adaptation for gradients. Muzeau et al. [77] observed similar results when testing trail running shoes with AFT foam compared with traditional running footwear. Although the effect of the AFT on oxygen consumption was more pronounced (+2.1%) in the flat condition, uphill running also indicated a ~1.0% RE improvement, whereas downhill running resulted in only a minor (+0.2%) improvement. By contrast, Hunter et al. [127] observed no metabolic benefits when comparing Saucony Pro (carbon-plated) to conventional shoes across 0%, +4%, and −4% inclines (~2.3°). They concluded that incline-specific adaptations may not be necessary, opposing the earlier findings of Whiting, Hoogkamer, and Kram [126].

Corbí-Santamaría et al. [117] further reported no RE improvement when using AFT on varied outdoor terrain. However, biomechanical adaptations were evident, including reduced step frequency and increased vertical oscillation, especially on inclines. Runners also perceived reduced forefoot flexibility in AFT models, suggesting that shoes optimised for flat roads may not translate to unstable surfaces like trails.

Overall, evidence regarding AFT effectiveness on gradients is inconsistent and strongly dependent on shoe model, running velocity, incline level, and individual response. This underscores the need for shoe designs adapted to varied surfaces to maximise AFT benefits.

### 9.5. The Role of Shoe Mass in Running Economy: Reevaluating Its Impact on AFT

Earlier studies consistently showed an inverse relationship between shoe mass and RE, with oxygen cost increasing by approximately 1% per additional 100 g [28,78,107]. Frederick [78] first reported this trend, and Franz et al. [28] confirmed nearly identical effects, observing VO_2_ increases of 0.92% (barefoot) and 1.19% (shod) per 100 g. Hoogkamer et al. [107] likewise reported a 1.11% increase in metabolic rate at 12.6 km/h and a 0.78% slower 3000 m time trial for each 100 g added to racing flats.

In AFT, however, mass interacts with advanced design features, making the classic 1% rule less predictive [33]. Cushioning and midsole material properties exert a major influence on locomotion economy [50,60,62]. Thicker cushioning layers can improve RE more than lighter weight alone, as added mass near the body’s centre of mass reduces moment of inertia and energetic cost [78,128]. Combined with longitudinal bending stiffness (LBS) and carbon plates, such cushioning enhances the conditions for reducing energy consumption [50].

Empirical evidence supports this. Joubert et al. [79] reported a 2% improvement in RE with AFT road shoes and spikes versus conventional models, with the heavier road shoes showing the greatest benefit. Barnes and Kilding [15] found that Nike Vaporfly (205 g) improved RE by 4.2 ± 1.2% compared with Adidas Adios Boost 3 (236 g) and by 2.6 ± 1.3% compared with Nike Matumbo spikes (118 g) at velocities of 14–18 km/h. Despite being 87 g heavier than the spikes, Vaporfly retained a 2.9 ± 1.3% RE advantage even after additional mass was added to match the Adidas shoe. Similarly, Hoogkamer et al. [14] reported ~4% lower metabolic cost for Vaporfly versus Adidas Adios Boost and Nike Zoom Streak 6 when shoe masses were equalised.

These results suggest that while shoe mass clearly influences conventional footwear, the effect cannot be directly applied to AFT, particularly road models introduced since 2017 [33]. The impact of shoe mass likely depends on both distance and velocity. At higher velocities, differences of up to 50 g appear negligible [14,103]. In contrast, over shorter distances, shoe mass may play a greater role, whereas in longer races, midsole properties are more decisive, facilitating energy return and sustaining economical technique over time [2,14].

### 9.6. Training Level, Racing Performance, and Individual Variability in Response to AFT

Individual response and training level are major factors influencing adaptation to AFT and the benefits gained. Identical shoe models can elicit highly individualised biomechanical responses [98], resulting in markedly different effects on RE [5,38]. This complexity makes it difficult to define the overall effect of AFT, particularly from a biomechanical standpoint [24,24].

Based on response, runners can be classified as non-responders, positive responders, or negative responders [33]. These differences are associated with natural RE, training level [5,50], individual biomechanics [98], and potentially shoe-specific biomechanical “skills” required to exploit a model’s full potential [25]. Gradual integration of AFT into training may further maximise benefits [93].

AFT often alters spatio-temporal variables, which can improve, reduce, or have no effect on RE depending on the runner [12,84]. For high-level athletes with already stable running biomechanics and efficient RE, improvements could be smaller, as their efficiency is close to physiological limits [5]. In contrast, recreational runners, whose gait patterns may be less stable, are more responsive to shoe-induced changes [50,129], but it does not always lead to a positive outcome (e.g., improved RE). Among recreational runners, RE improvements often correlate with subjective comfort, with greater RE gains observed in shoes rated more comfortable [119].

Transitioning to new footwear, however, can present risks. Eken et al. [85] found that runners reported higher discomfort and injury incidence when switching to cushioned shoes (On CloudSurfer) compared with their habitual footwear during an eight-week transition. Biomechanical changes induced by AFT may, therefore, enhance or impair performance depending on how well an individual’s biomechanics align with the shoe’s design. Joubert and Jones [33], for example, compared seven carbon-plated models and reported that fewer than 10% of runners shifted fully to a forefoot strike pattern. The lowest responder in Nike Alphafly already exhibited high natural RE, with cadence averaging 186 steps/min and vertical oscillation ~8.5 cm, compared with higher-responding runners who had lower cadence and greater oscillation.

Rodrigo-Carranza et al. [50] compared carbon-plated shoes with increased LBS to control models in trained and national-level runners. At both submaximal intensities (9–13 km/h) and in 3000 m time trials, trained runners showed greater improvements than national-level athletes. Among national-level runners, improvements were observed only at high velocities, again attributed to already elevated baseline RE.

Knopp et al. [5] compared three carbon-plated shoes and racing flats in world-class Kenyan and amateur European runners at ~70–75% “VO_2_”max (marathon pace). Amateur runners demonstrated RE changes ranging from +9.7% to −1.1%, while Kenyan runners ranged from +11.4% to −11.3%. Barnes and Kilding [15] likewise found substantial inter-individual variability, with RE differences of 1.72–7.15% between Nike and Adidas models and 0.50–5.34% between Nike road shoes and spikes.

These findings indicate that despite training level or genetic advantages, large inter-individual variations exist in response to AFT [98]. It cannot be unequivocally stated that AFT guarantees improvements in RE or biomechanics, nor are benefits evenly distributed across runners [25]. Runners whose biomechanics align more closely with a shoe’s design “sweet spot” have greater potential to benefit, which also explains why elite athletes often use prototypes customised to their biomechanical, anthropometric, and physiological characteristics.

### 9.7. AFT and Fatigue Resistance

A growing body of research has linked AFT to improved neuromuscular fatigue resistance in long-distance running. These effects suggest that AFT may not only help maintain performance but also delay fatigue-related declines in RE, offering potential benefits for endurance athletes beyond immediate performance.

Ruiz-Alias et al. [130] tested thirteen highly trained athletes in 9-minute and 3-minute time trials using Nike ZoomX Dragonfly track spikes and Nike ZoomX Vaporfly Next% 2 road shoes. Although pace differences were not statistically significant (p≥0.072), runners in AFT shoes increased pace in the final lap, unlike those in spikes. Ground contact time decreased across the session, and stride length tended to increase with AFT. Most notably, neuromuscular fatigue was lower: countermovement jump height decreased by −5.6% in spike users compared with only −0.61% in AFT users. These findings suggest that AFT may help sustain propulsion efficiency and cushioning during prolonged running, enhancing fatigue tolerance.

Similarly, Castellanos-Salamanca et al. [16] compared Vaporfly Next/textpercent 2 shoes with conventional models during interval training (5 × 1000 m, 90 s recovery). AFT shoes improved training performance by 2.4% (*p* = 0.009) without significant changes in heart rate or running power and were associated with reduced neuromuscular fatigue and perceived muscle pain. Countermovement jump height decreased less following the AFT session, supporting its potential role in attenuating fatigue. In agreement, Kirby et al. [67] reported that recreational runners wearing Vaporfly 4% shoes experienced less post-marathon muscle soreness and muscle damage compared with conventional footwear.

Beyond fatigue outcomes, Xu et al. [48] examined how carbon-plate geometry influences biomechanics under fatigue. Compared with flat plates, curved plates reduced hip and knee contact angles, decreased hip flexion moments, and modified tibialis anterior activation patterns both before and after fatigue exposure. These adaptations may contribute to maintaining efficiency and lowering musculoskeletal load under fatigue.

### 9.8. The Role of Foot Strike Patterns

Foot strike patterns can considerably alter running kinematics, thereby influencing RE and overall efficiency. AFT is often associated with biomechanical adaptations, as runners adjust their gait to the shoe’s mechanical features. Foot strike-related kinematic changes may positively, negatively, or neutrally affect RE. No single running style has been proven superior with AFT, although emerging evidence points to potential trends warranting further investigation.

Hoogkamer et al. [14] compared the Nike Zoom Vaporfly prototype with traditional marathon shoes (Nike Zoom Streak 6) and the then–world record model, Adidas Adizero Adios BOOST 2. Energy consumption did not differ significantly among heel, midfoot, or forefoot strikers. However, a borderline shoe–foot strike interaction (*p* = 0.0502) suggested slightly greater benefits for dominant rearfoot strikers.

A similar interaction is seen with sole stiffness [30,54]. McLeod et al. [54] tested six custom-made carbon fibre soles of varying stiffness at lower (2.98 m/s) and higher (4.47 m/s) velocities. Heel strikers benefited the most from greater stiffness at higher intensities, likely due to kinematic differences that placed them in a more favourable position than midfoot strikers. Terrain may also modulate these effects. Fukuchi et al. [131] tested AFT trail shoes with an inserted carbon sole and found that the curved plate design produced greater forefoot benefits during uphill running, where forefoot loading is naturally increased.

Martinez et al. [96] further analysed super shoes (Nike Vaporfly Next% 2) in relation to foot strike pattern, reporting a 4.2% improvement in metabolic power compared with control shoes. Importantly, no significant interaction emerged between foot strike pattern and the metabolic benefit.

At present, relatively few studies treat foot strike as a primary determinant of AFT effectiveness. Instead, most mention foot strike as a possible secondary factor or hypothesis, inferred from kinematic and RE data. To establish more objective patterns, further dedicated research is required.

### 9.9. Sex Differences in Biomechanical and Performance Outcomes with AFT

When evaluating the influence of AFT on RE and performance, it is essential to consider sex differences. Anatomical and physiological distinctions can influence biomechanical parameters during running and may shape the extent to which athletes benefit from AFT footwear.

Since the release of AFT in 2017, performance improvements have been particularly notable in women across distances from 1500 m to the marathon [19,20,21,22,23]. Analyses of elite performances show that women improved by ~1.7–2.3% compared with 0.6–1.5% in men, corresponding to ~2 min faster marathon times [19]. Similar findings have been reported by Langley et al. [21], who observed ~1.7% gains across 10 km, half-marathon, and marathon in the fastest female runners during the AFT era, with greater improvements in AFT adopters compared with controls. Willwacher et al. [23] summarised and systematically analysed the 100 fastest outdoor track and road race performances for men and women from 2010 to 2022. The performance improvements were more pronounced in women and in longer-distance events (above 1500 m). In contrast, men exhibited the greatest improvements in events exceeding 5000 m. Senefeld et al. [20] further showed that among the top 50 male and female finishers in World Marathon Majors, women improved by 4.3 min compared with men’s 2.8 min, with relative improvements of 1.6% versus 0.8%. Overall, these gains have contributed to narrowing the performance gap between men and women from ~12% to ~8–9% over long-distance events [22].

Several factors may underlie these observed differences. One explanation relates to physiological reserve and training history. Men’s performances have historically clustered more closely to physiological ceilings, whereas women may retain greater room for improvement [22].

Intensity levels and velocity thresholds may also be important [23]. Some evidence suggests that AFT benefits manifest most clearly above certain running velocities, while studies at lower velocities and in recreational runners report more modest or inconsistent effects [38,95,113,115]. This may be linked to velocity-dependent alterations in running biomechanics [114].

Finally, biomechanical and anthropometric factors can influence the benefits of AFT [23]. Energy-saving effects depend on compressing the foam midsole and flexing the sole during stance. Carbon plates with greater stiffness require substantial muscle force to deform the midsole and fully use the “shoe potential”. Studies on midsole stiffness, even before carbon plates were introduced, show that heavier and stronger athletes gain greater metabolic savings due to enhanced foam compression [70]. For example, Roy and Stefanyshyn [43] reported ~1% RE improvements with stiffer midsoles, with heavier runners benefiting more. Oh and Park [70] also observed an inverse correlation between body mass and “VO_2_” change in shoes with medium stiffness.

Although direct evidence for AFT-specific footwear is limited, these observations suggest that body mass and strength may be among the main explanations for the broadly observed sex differences. Future research should analyse these factors more deeply, with the aim of clarifying whether anatomical, physiological, or biomechanical differences underpin the observed patterns.

### 9.10. The Evolving Role of AFT in Trail and Mountain Running Biomechanics and Performance

The popularity of trail running has grown significantly in recent years, and an increasing number of running shoe manufacturers now offer AFT specifically designed for trail conditions. However, evidence on their effectiveness remains limited, making it difficult to evaluate the role of AFT in trail performance.

Trail running, by contrast, takes place in natural environments with diverse terrains, such as mountains, slopes, meadows, and forests, with asphalt typically representing only ~20% of total distance [131,132]. Such surface differences substantially alter running biomechanics, meaning that results from asphalt cannot be directly extrapolated to trails [133,134]. Open questions remain as to whether the design of AFT shoes, often characterised by minimal ankle and foot joint stabilisation, can be reconciled with the stability and containment requirements of trail running and whether including these support functions could allow AFT to deliver comparable improvements to those observed on asphalt.

Several models of carbon-plated trail shoes with modified outsole surfaces and added tread are now available, but most retain very light and thin upper constructions. Moreover, the increased stack height typical of carbon shoes is known to reduce stability and may even increase injury risk on uneven surfaces (see Section 4.3, Stack height; [126]). This likely contributes to the absence of consistent performance benefits in trail running, although biomechanical effects have been observed [117], similar to those reported in road-running AFT studies [42]. Another limiting factor is the scarcity of empirical data, as trail-specific AFT is still relatively new. In addition, laboratory-based performance testing is almost impossible under trail conditions. While treadmill running may help predict potential trends, it cannot replicate natural trail environments.

#### Current Research Findings in Trail Running

To the authors’ knowledge, only a few studies have examined the impact of carbon-plated shoes on RE under natural trail conditions.

Fukuchi et al. [131] compared trail-specific shoes (Carbitex Speedland—considered the world’s first hyper-performance trail running shoe, combining high-quality materials with comfort and sustainability [135]) with and without carbon soles in eleven runners. Testing was performed over a 50 m marked section including ~18.5% ascent and ~17.2% descent, running at self-selected comfortable velocity for 10 min each. Segment acceleration and plantar pressure were assessed using IMU sensors. No significant differences were found in velocity, contact time, foot acceleration, or maximum plantar pressure across the four-foot regions (toes, metatarsals, midfoot, and heel). However, carbon-plated shoes produced lower maximum axial acceleration of the lower leg during uphill running and slightly higher forefoot pressure. This likely reflects biomechanical adaptations, as uphill running requires a forward lean and increased forefoot loading [127].

The authors proposed that this forefoot shift may enhance the spring-like effect of the carbon sole. Specifically, the curved plate increases LBS, limits foot flexion, and promotes energy savings [30]. Consequently, AFT for trail running may be more effective uphill than downhill, since forefoot activation is more pronounced during uphill running [127]. A limitation of the study was the relatively low running velocity (2.5 m/s) and short uphill/downhill distances (11 m), which may not have allowed the shoes to demonstrate their full potential [38].

A recent study by Corbí-Santamaría et al. [117] supports these observations. Although AFT did not improve simulated mountain running performance or physiological responses, it significantly altered biomechanics, reducing step frequency and increasing vertical oscillation of the centre of mass, particularly on variable terrain.

Finally, one of the earliest studies assessing physiological variables reported a ~1% improvement in RE and reduced perceived effort when running in carbon-plated shoes compared with traditional footwear across different gradient conditions, with the most pronounced effect observed on flat terrain [77]. However, a key limitation of the study was that all trials were conducted on a treadmill, which cannot accurately be translated into real-life trail running conditions [89].

Despite the limited and mixed evidence, manufacturers continue to develop AFT models for trail running. More comprehensive studies are required to identify the optimal design features that balance stability and support while maximising potential RE benefits under the unique biomechanical demands of trail environments.

## 10. The ‘Barefoot Running Era’: Lessons for Modern Running Technology

The barefoot and minimalist running trend peaked in the late 2000s, driven by claims of reduced injury risk, natural gait promotion, and improved RE. Although this movement has since declined, it represents an important “zero point” in the evolution of running footwear and provides valuable lessons for understanding the mechanisms and performance outcomes of modern AFT, particularly in relation to differences in gait mechanics, loading rates, shoe mass, and individual variability.

Often associated with reduced vertical ground reaction force loading rates (LRs), barefoot running has been proposed as more economical than shod running [136,137]; however, individual factors should be considered [138]. Tam et al. [139] reported that habitual shod runners displayed a 54% higher average LR when running barefoot, with wide inter-individual ranges (12.3–622.8 BW/s barefoot vs. 27.2–315.3 BW/s shod). This counterintuitive result highlights that benefits such as reduced LR only emerge when runners successfully adapt to a mid-/forefoot strike; non-responders who maintain rearfoot striking derive little advantage and may even increase injury risk [140,141,142]. Many less trained runners cannot perform rapid gait adjustments due to insufficient muscular conditioning [138], often resulting in foot, calf, or Achilles pain [6,142]. Similar adaptation is also required when running in AFT: despite the design element—carbon fibre plate, foam, stack height, and toe spring— and the ability to reduce energy dissipation, their effectiveness depends heavily on the runner’s training status, lower-limb muscle strength, and ability to adjust stride mechanics to fully exploit the potential provided by the shoes [98,143]. In this context, the need for an adaptation period and gradual integration of AFT into training is critical to maximising performance outcomes while simultaneously reducing potential injury risks, particularly among less trained runners or those with insufficient muscular conditioning [93].

Claims that barefoot running enhances intrinsic foot muscle stiffness remain inconclusive, with some evidence suggesting greater stiffness under shod conditions [144,145,146]. On the other hand, barefoot running is associated with greater pre-activation of the triceps surae [147]. AFT modifies load distribution differently: increased longitudinal bending stiffness and carbon plates reduce excessive metatarsophalangeal dorsiflexion, lowering energy absorption and dissipation [30]. Thus, while barefoot running demands greater muscular conditioning to tolerate higher ankle loads, AFT reduces muscular effort during the push-off phase, highlighting a shift from reliance on intrinsic adaptation to reliance on engineered support.

Minimalist designs emphasised reduced cushioning, lower stack height and more natural gait, emphasising the role of shoe mass in determining RE [28,78,148]. Contrary to that, AFT is more focused on specific design element integration and their combinations [33,37,61], which marks a paradigm shift: where minimalist shoes sought efficiency through natural adaptation and lighter mass, AFT pursues efficiency through engineered energy-management systems, largely independent of shoe mass.

Together, these historical and modern approaches illustrate the evolution from reliance on natural adaptation to reliance on technological innovation in shaping running economy and performance. Looking ahead, integrating these lessons underscores that future directions in running footwear must balance technological innovation with individual adaptation capacity, ensuring that advances in design translate into sustainable performance gains and reduced injury risk across diverse runner populations.

## 11. Conclusions

Despite extensive study of AFT in recent years, important misconceptions and uncertainties remain regarding its working mechanisms and performance benefits. Much of this stems from methodological limitations, including (1) difficulty in explaining how individual shoe components contribute to overall improvements in RE and performance, (2) challenges in linking biomechanical changes to metabolic outcomes, (3) over-reliance on group means rather than individual differences, and (4) the predominance of treadmill-based testing rather than field studies. Nevertheless, several clear insights can be drawn from the collected evidence, along with directions for future research:

1. The carbon fibre plate is not the sole performance determinant; improvements result from the combined interaction of multiple design features and should be evaluated holistically.
2. Current “super spikes” provide smaller performance gains than AFT road shoes, likely due to lower cushioning and reduced shock absorption despite their lighter mass.
3. AFT primarily influences biomechanics rather than physiological parameters, with the greatest effects being observed on stride mechanics, contact time, and force application, particularly at higher speeds.
4. Efficiency must be balanced with injury risk. While AFT can improve speed and economy, long-term effects on musculoskeletal health are unclear. High stack height with low weight may reduce stability and increase injury risk, especially in runners with weakness, poor control, or excessive pronation.
5. Velocity influences the magnitude of benefits. Submaximal speeds close to race pace tend to maximise AFT effects and comfort, whereas improvements are smaller at lower speeds due to mechanical constraints.
6. Future research should prioritise the integration of field-based wearable sensor data with AI-driven biomechanical modelling, enabling analyses that more accurately reflect real-world running conditions while preserving natural and comfortable movement patterns.
7. Shoe mass has a limited influence. The traditional rule of a 1% increase in oxygen cost per 100 g does not fully apply to AFT; heavier shoes with advanced features can still outperform lighter, less advanced models.
8. Individual adaptation is crucial. The same AFT model can produce different running economy effects across runners, depending on biomechanical compatibility and the ability to utilise specific movement patterns.
9. Recreational runners also benefit from AFT, though to a lesser extent than elite athletes and only when their biomechanics align with the shoe’s functional design.
10. Performance gains may be reduced on trail surfaces. Technologies designed for asphalt do not consistently transfer to uneven terrain, highlighting the need for trail-specific AFT development.

## 12. Practical Implications

These findings highlight the importance of a personalised approach, gradual integration, and fair-use boundaries in the application of advanced footwear technology (AFT). Athletes should select footwear models that align with their individual anthropometric and biomechanical parameters, as well as their physical capacity, to effectively adapt to the shoe-induced mechanisms. Progressive integration of AFT into training is necessary to allow the musculoskeletal system to adapt—high-performance models should be reserved for racing or specific sessions to balance efficiency gains with long-term durability and injury prevention. Finally, running velocity should be carefully considered and strategically leveraged when selecting AFT, as it determines both performance outcomes and the ability to fully utilise the shoe’s potential.

## 13. Future Directions

More studies are needed to clarify the long-term health effects of AFT, particularly its influence on musculoskeletal function and injury risk with sustained use. Future research should focus on more field-based studies using IMUs as the primary measurement tools and AI-driven biomechanical modelling. Standardised research protocols (e.g., same-day repeated RE trials, velocity selection tied to individual race pace, and accounting for inter-individual variability) could further enhance the validity and representativeness of the data. Furthermore, investigations into prolonged running distances at velocities close to or equivalent to race pace are important, as they can reveal how AFT influences fatigue resistance and running performance under competitive conditions. Research should also explore trail-specific AFT designs and durability testing to guide both competitive and recreational applications of this rapidly evolving technology.

## Figures and Tables

**Table 1:** Summary of World Athletics Footwear Regulations (2020). Maximum stack height, plate count, and availability requirements categorised by Advanced Footwear Technology type: road racing shoes and track spikes.

Footwear Type	Maximum Stack Height	Plate Count	Availability Requirements
Road racing shoes	40 mm	Maximum of one plate or similar device	Must be available for purchase at least four months before competition
Track spikes	30 mm	One functional plate permitted	Must comply with the four-month commercial availability requirement
NCAA and high school (USA)	Not restricted unless regulated by WA	No WA plate restriction	No WA availability requirement

## Data Availability

No new data were created or analyzed in this study.
